# Supplementary material for: Hierarchically structuralized hydrogels with ligament-like mechanical performance
Source: Nat Commun. 2025 Dec 13;16:11492. doi: 10.1038/s41467-025-66536-8 (PMC12749280; doi:10.1038/s41467-025-66536-8)
Supplement: Supplementary file 1 — Supplementary Information [file 41467_2025_66536_MOESM1_ESM.pdf]

Supplementary Information

Hierarchically structuralized hydrogels with ligament-like mechanical performance

*Diwei Shi[1,†], Donghwan Ji[2,3,†], Jinhye Bae[1,2,3]**

[1] Materials Science and Engineering Program, University of California San Diego, La Jolla, CA 92093, United States

[2] Aiiso Yufeng Li Family Department of Chemical and Nano Engineering, University of California San Diego, La Jolla, CA 92093, United States

[3] Department of Chemical Engineering, Chung-Ang University, 84 Heukseok-ro, Dongjak-gu, Seoul 06974 Republic of Korea

†These authors contributed equally.

*Corresponding authors: *J. Bae* (jbae@cau.ac.kr)

Publications	Elastic modulus (MPa)	Fracture tensile strength (MPa)	Fracture elongation (%)	Work-of-fracture (MJ m⁻³)
This work	131	61.5	430	135
Anisotropically Fatigue-Resistant Hydrogels, **Advanced Materials (2021)**	1	2.5	430	
Strong fatigue-resistant nanofibrous hydrogels inspired by lobster underbelly, **Matter (2021)**	5	3.5	340	
Poly(vinyl alcohol) Hydrogels with Broad-Range Tunable Mechanical Properties via the Hofmeister Effect, **Advanced Materials (2021)**	2.5	15	2100	153
Strong tough hydrogels via the synergy of freeze-casting and salting out, **Nature (2021)**	25	23.5	1400	210
Hierarchical Multiscale Hydrogels with Identical Compositions Yet Disparate Properties via Tunable Phase Separation, **Advanced Functional Materials (2022)**	1.1	7	1600	
Super-Strong, Nonswellable, and Biocompatible Hydrogels Inspired by Human Tendons, **ACS Applied Materials & Interfaces (2022)**	35.6	19.3	300	32.1
Strong Tough Conductive Hydrogels via the Synergy of Ion-Induced Cross-Linking and Salting-Out, **Advanced Functional Materials (2022)**	3.7	15	770	56
Bioinspired 2D Isotropically Fatigue-Resistant Hydrogels, **Advanced Materials (2022)**	18	9	250	
Coordinatively Stiffen and Toughen Hydrogels with Adaptable Crystal-Domain Cross-Linking, **Advanced Materials (2023)**	52.3	28	1200	199
Solvent-Exchange-Assisted Wet Annealing A New Strategy for Superstrong, Tough, Stretchable, and Anti-Fatigue Hydrogels, **Advanced Materials (2023)**	2.7	11.2	1360	82.9
Reinforcing Hydrogel by Nonsolvent-Quenching-Facilitated In Situ Nanofibrosis, **Advanced Materials (2023)**	0.6	2.7	700	10.4
Toughening Double-Network Hydrogels by Polyelectrolytes, **Advanced Materials (2023)**	0.5	1.5	750	5.4
A Biomimetic "Salting Out—Alignment—Locking" Tactic to Design Strong and Tough Hydrogel, **Advanced Materials (2024)**	7.5	20	600	70.7
Tough Supramolecular Hydrogels Crafted via Lignin-Induced Self-Assembly, **Advanced Materials (2024)**	14	20	500	50
Bioinspired structural hydrogels with highly ordered hierarchical orientations by flow-induced alignment of nanofibrils, **Nature Communications (2024)**	5	14	1700	154
3D printable strong and tough composite organo-hydrogels inspired by natural hierarchical composite design principles, **Nature Communications (2024)**	20.3	6.9	350	17.5
Spider-silk-inspired strong and tough hydrogel fibers with anti-freezing and water retention properties, **Nature Communications (2024)**	36.8	24.4	850	162
Control nucleation for strong and tough crystalline hydrogels with high water content, **Nature Communications (2024)**	1.9	7.4	1350	
Bamboo-inspired ultra-strong nanofiber-reinforced composite hydrogels, **Nature Communications (2025)**	35	48	470	122.4
Hierarchically aligned heterogeneous core-sheath hydrogels, **Nature Communications (2025)**	6.8	55.3	3300	1031
Cartilage-bioinspired tenacious concrete-like hydrogel verified via in-situ testing, **Nature Communications (2025)**	3.78	10.5	260	6.8
Robust super-structured porous hydrogel enables bioadaptive repair of dynamic soft tissue, **Nature Communications (2025)**	32.4	22.2	400	61.9
Thermoplastic Elastomer-Reinforced Hydrogels with Excellent Mechanical Properties, Swelling Resistance, and Biocompatibility, **Advanced Science (2025)**	2.8	11.2	1030	62.7
Muscle-Inspired Self-Growing Anisotropic Hydrogels with Mechanical Training-Promoting Mechanical Properties **Advanced Materials (2025)**	2.85	14.1	5700	465

Supplementary Table 1. Summary of elastic modulus, fracture tensile strength, fracture strain, and work-of-fracture (toughness) of previously reported mechanically reinforced PVA-based hydrogels. Bright blue square: published in 2021; brown rhombus-shape diamond: published in 2022; green circle: published in 2023; purple triangle: published in 2024; Dark blue circle: published in 2025. The strength and elastic modulus of natural ligaments were displayed in a range based on literature, but the toughness value was not easy to be specifically displayed.

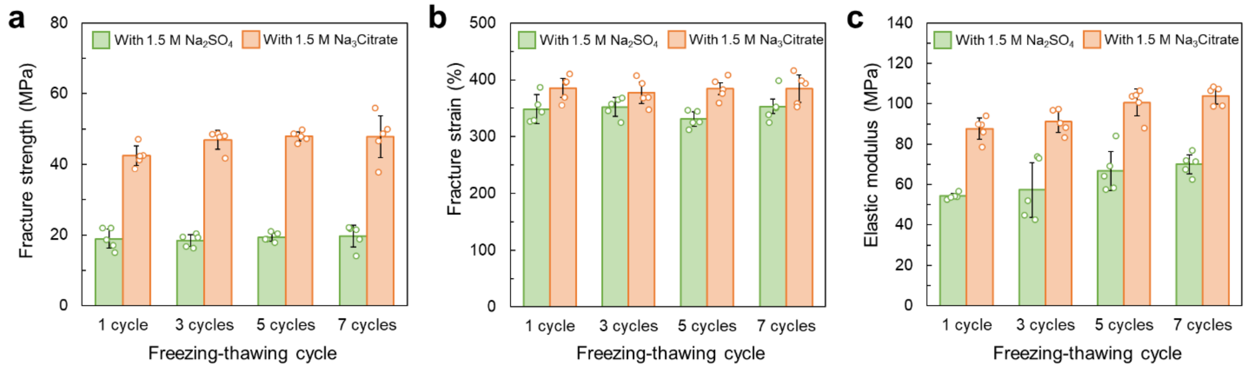

Supplementary Fig. 1. (a) Fracture strength, (b) fracture strain, and (c) elastic modulus of PVA-NC hydrogel fibers prepared by varying freezing-thawing cycles and treatment with 1.5 M Na_2SO_4 and 1.5 M Na_3Citrate, respectively. Data are presented as mean ± standard deviation (n = 5).

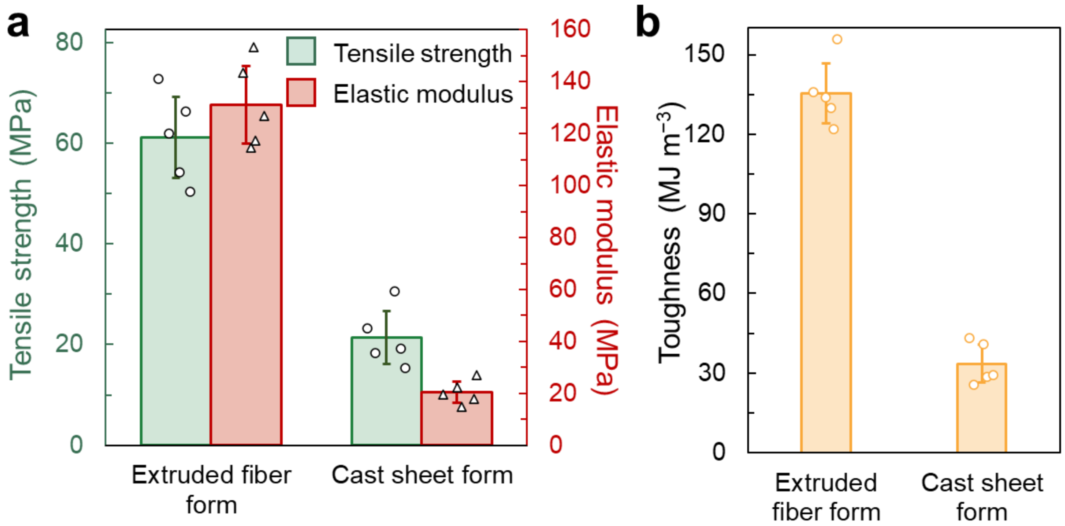

Supplementary Fig. 2. (a) Fracture tensile strength and elastic modulus, and (b) toughness of PVA-NC/FT/S hydrogels in extruded fiber form and cast sheet form, respectively. The hydrogels were processed by a single FT cycle and treated with 2.8 M Na_3Citrate solution. Data are presented as mean ± standard deviation (n = 5).

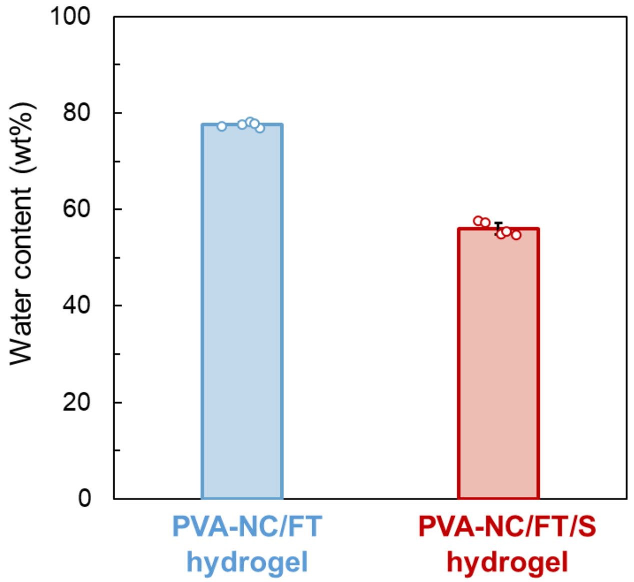

Supplementary Fig. 3. Water content of two different hydrogels. Data are presented as mean ± standard deviation (n = 5).

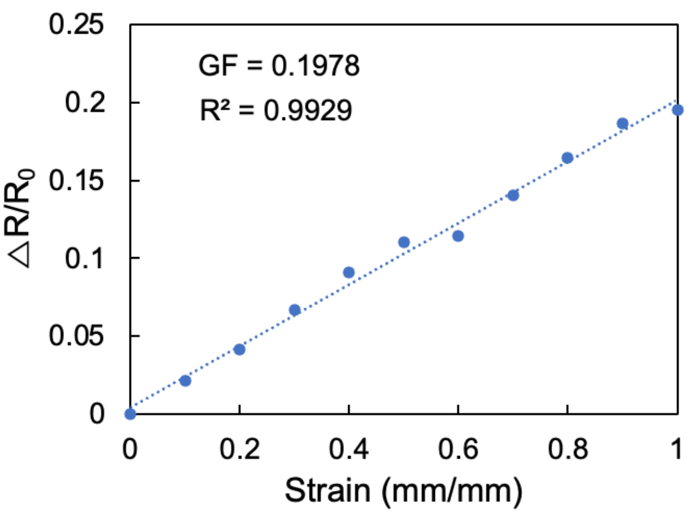

Supplementary Fig. 4. The relative resistance variation ($\Delta R/R_0$) of 8-fiber-braided hydrogel bundle over a strain region of 0–100%. The gauge factor (GF), which is defined as the slope of the $\Delta R/R_0$ versus applied strain, is 0.1978.

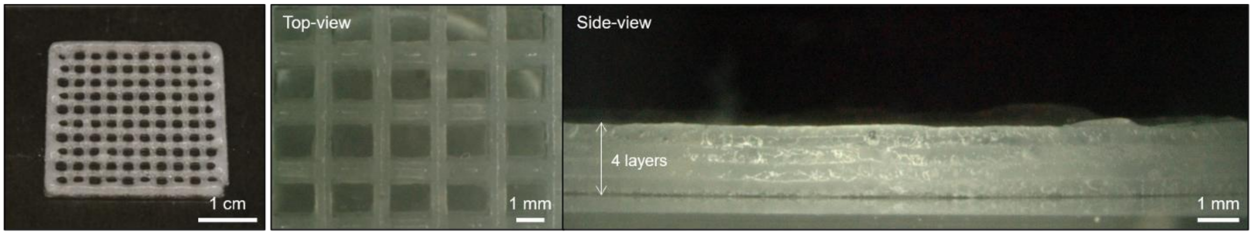

Supplementary Fig. 5. Photographs of the printed structure of PVA-NC/FT/S hydrogel.

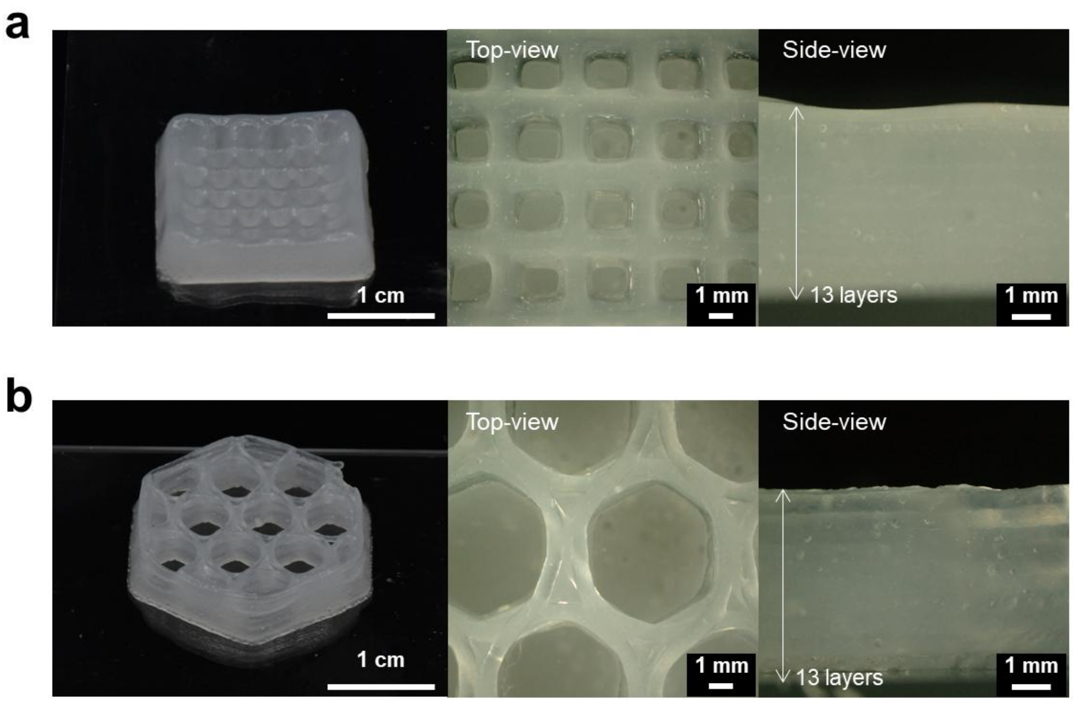

Supplementary Fig. 6. Photographs of the printed structures of PVA-NC/FT/S hydrogel. (a) Square structure printed by a grid printing route. (b) Hexagon structure printed by a honeycomb printing route.

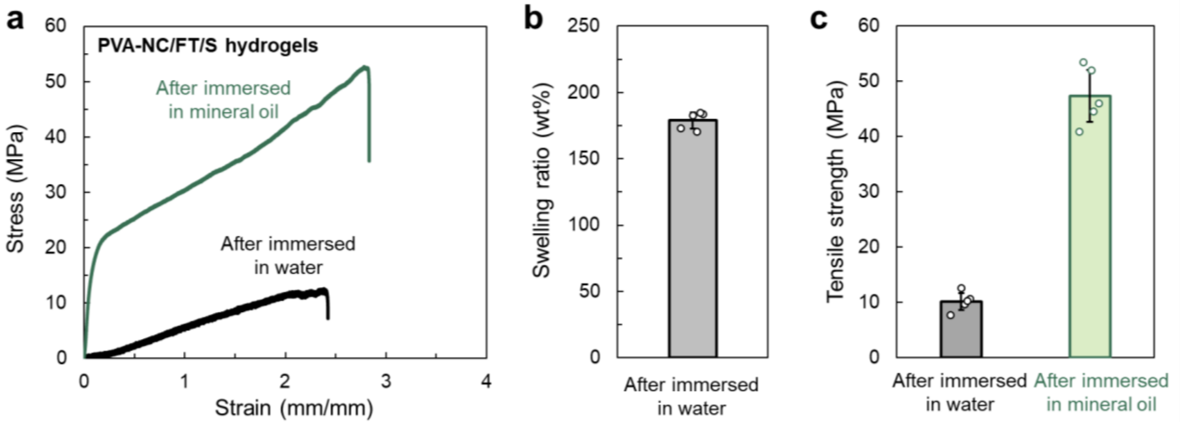

Supplementary Fig. 7. (a) Stress-strain curves of PVA-NC/FT/S hydrogel after immersed in mineral oil and in pure water, respectively. (b) Swelling ratio of the hydrogel in pure water. Data are presented as mean ± standard deviation (n = 5). (c) Tensile strength of two different hydrogels. Data are presented as mean ± standard deviation (n = 5).

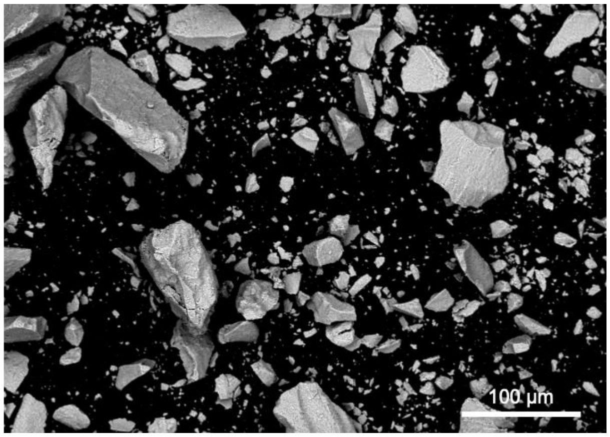

Supplementary Fig. 8. SEM image of as-received dry NC powder.

Supplementary Movie 1. Loading (404.5g) test of individual PVA-NC hydrogel fiber treated with one freezing-thawing cycle and 2.8 M Na_3Citrate salting-out processes.

Supplementary Movie 2. Loading (404.5g) test of individual PVA-NC/FT/S, PVA-NC/S and PVA-NC/FT hydrogel fiber.

Supplementary Movie 3. Loading (13.6 kg) test of 50-fiber-braided hydrogel bundle.

Supplementary Movie 4. Real-time printing process of PVA-NC mixture.
