# Supplementary material for: Hierarchically structuralized hydrogels with ligament-like mechanical performance
Source: Nat Commun. 2025 Dec 13;16:11492. doi: 10.1038/s41467-025-66536-8 (PMC12749280; doi:10.1038/s41467-025-66536-8)
Supplement: Supplementary file 2 — Description of Additional Supplementary Files [file 41467_2025_66536_MOESM2_ESM.pdf]

Description of Additional Supplementary Files

Supplementary Movie 1. Loading (404.5g) test of individual PVA-NC hydrogel fiber treated with one freezing-thawing cycle and 2.8 M Na_3Citrate salting-out processes.

Supplementary Movie 2. Loading (404.5g) test of individual PVA-NC/FT/S, PVA-NC/S and PVA-NC/FT hydrogel fiber.

Supplementary Movie 3. Loading (13.6 kg) test of 50-fiber-braided hydrogel bundle.

Supplementary Movie 4. Real-time printing process of PVA-NC mixture.
